# Structure-Based Design of Nipah Virus Vaccines: A Generalizable Approach to Paramyxovirus Immunogen Development

**DOI:** 10.3389/fimmu.2020.00842

**Published:** 2020-06-11

**Authors:** Rebecca J. Loomis, Guillaume B. E. Stewart-Jones, Yaroslav Tsybovsky, Ria T. Caringal, Kaitlyn M. Morabito, Jason S. McLellan, Amy L. Chamberlain, Sean T. Nugent, Geoffrey B. Hutchinson, Lisa A. Kueltzo, John R. Mascola, Barney S. Graham

**Affiliations:** ^1^Viral Pathogenesis Laboratory, Vaccine Research Center, National Institute of Allergy and Infectious Diseases, National Institutes of Health, Bethesda, MD, United States; ^2^Virology Laboratory, Vaccine Research Center, National Institute of Allergy and Infectious Diseases, National Institutes of Health, Bethesda, MD, United States; ^3^Electron Microscopy Laboratory, Cancer Research Technology Program, Leidos Biomedical Research, Inc., Frederick National Laboratory for Cancer Research, Frederick, MD, United States; ^4^Vaccine Production Program, Vaccine Research Center, National Institute of Allergy and Infectious Diseases, National Institutes of Health, Bethesda, MD, United States; ^5^Department of Molecular Biosciences, The University of Texas at Austin, Austin, TX, United States

**Keywords:** Nipah virus, stabilized prefusion F, structure-based vaccine design, G attachment protein, pre-F/G chimeric immunogen, pandemic preparedness

## Abstract

Licensed vaccines or therapeutics are rarely available for pathogens with epidemic or pandemic potential. Developing interventions for specific pathogens and defining generalizable approaches for related pathogens is a global priority and inherent to the UN Sustainable Development Goals. Nipah virus (NiV) poses a significant epidemic threat, and zoonotic transmission from bats-to-humans with high fatality rates occurs almost annually. Human-to-human transmission of NiV has been documented in recent outbreaks leading public health officials and government agencies to declare an urgent need for effective vaccines and therapeutics. Here, we evaluate NiV vaccine antigen design options including the fusion glycoprotein (F) and the major attachment glycoprotein (G). A stabilized prefusion F (pre-F), multimeric G constructs, and chimeric proteins containing both pre-F and G were developed as protein subunit candidate vaccines. The proteins were evaluated for antigenicity and structural integrity using kinetic binding assays, electron microscopy, and other biophysical properties. Immunogenicity of the vaccine antigens was evaluated in mice. The stabilized pre-F trimer and hexameric G immunogens both induced serum neutralizing activity in mice, while the post-F trimer immunogen did not elicit neutralizing activity. The pre-F trimer covalently linked to three G monomers (pre-F/G) induced potent neutralizing antibody activity, elicited responses to the greatest diversity of antigenic sites, and is the lead candidate for clinical development. The specific stabilizing mutations and immunogen designs utilized for NiV were successfully applied to other henipaviruses, supporting the concept of identifying generalizable solutions for prototype pathogens as an approach to pandemic preparedness.

## Highlights

- Structure-guided stabilization of Nipah virus prefusion F glycoprotein trimers.
- Chimeric proteins composed of Nipah virus pre-F trimer linked to 3 Nipah virus G monomers induce potent neutralizing activity targeting both F and G.
- Vaccine antigens developed for other henipaviruses using Nipah virus design as prototype.

## Introduction

Nipah virus (NiV), an enveloped, non-segmented negative-strand RNA virus, is classified in the Henipavirus genus of the *Paramyxoviridae* family, along with closely related Hendra (HeV) and Cedar (CedPV) viruses, and several other uncharacterized henipaviruses isolated from Africa (1–7). NiV was first isolated during an outbreak on the Malaysian peninsula with 265 suspected infections and 105 deaths and another 11 infections and one death in Singapore that occurred between September 1998 and June 1999. Pigs were the apparent source of infection in the first outbreak with more than one million being culled (1, 8, 9). The Malaysian strain of NiV is primarily encephalitic with no documented cases of human-to-human transmission (10). Since its emergence, NiV has reappeared almost annually in outbreaks in Bangladesh and India often associated with a high mortality rate (60–70%) (11–17). While most cases have zoonotic exposures, the Bangladesh strain of NiV can also spread human-to-human by the respiratory route (12, 18–22), infection can be neurotropic, and patients often develop encephalitis (8, 15, 23–26). There is limited genomic variation between the two predominant strains of NiV, sharing ∼92% nucleotide homology (14).

Even though most outbreaks have been confined to Bangladesh and India, the natural reservoir of NiV appears to be fruit bats of the *Pteropodidae* family (27–29) from which NiV has been isolated throughout Southeast Asia. NiV also has a broad species tropism and can cause disease in horses and other domestic animals beyond pigs which expands the chances of zoonotic transmission from intermediate hosts (1, 13, 30–36). NiV is classified as a Biological Safety Level 4 (BSL 4) pathogen, considered a pandemic threat and listed as a high priority pathogen for intervention development by the World Health Organization (WHO), Centers for Disease Control and Prevention (CDC), and the Coalition for Epidemic Preparedness Innovations (CEPI) (37). The large zoonotic reservoir, potential for human-to-human transmission, and high fatality rate from henipavirus infections suggest a general paramyxovirus or henipavirus vaccine antigen design strategy is needed to prepare for future outbreaks.

All members of the *Paramyxoviridae* and *Pneumoviridae* have two membrane glycoproteins involved in receptor binding and viral entry, the attachment (G, H, or HN) and fusion (F) proteins, respectively (38), making them ideal targets for neutralizing antibodies (39). Paramyxoviruses and Pneumoviruses utilize a class I fusion glycoprotein that transitions between a metastable prefusion (pre-F) conformation and a stable postfusion (post-F) conformation to merge viral and cellular membranes (40–44). The crystal structure of prefusion NiV F was determined and adopts a similar overall architecture to parainfluenza virus prefusion F trimer structures (45–47). The protein folding patterns and subdomains of the prefusion NiV F trimer are similar to the F glycoprotein of respiratory syncytial virus, a Pneumovirus with a distinct metastable prefusion F glycoprotein conformation, which has been stabilized in the prefusion conformation by structure-based vaccine design (48, 49). RSV F stabilized in its prefusion conformation can induce high levels of RSV-neutralizing activity in humans and protection from RSV challenge in animal models (48, 50). The NiV G protein is a Type II membrane protein that facilitates attachment of NiV virions to target host cell membranes via ephrin B2/B3 receptors, and has a native tetrameric (dimer of homodimers) structure (51–56).

Several approaches have been applied for development of henipavirus interventions which have largely focused on the surface glycoproteins, G and F. The first, utilizes a recombinant subunit vaccine (HeV sG) that has been shown to protect against both HeV and NiV challenge in rabbits (57), ferrets (58, 59) and African green monkeys (AGMs) (60, 61). The HeV sG vaccine is currently used as a veterinary vaccine for HeV in horses (Equivac HeV, Zoetis) in Australia (58, 62–64) and is being considered as a human vaccine against NiV. The second, utilizes viral vectors such as canarypox encoding NiV F or NiV G (65), vaccinia viruses encoding both NiV F or NiV G (66), recombinant AAV encoding NiV G (67), recombinant rhabdoviruses (VSV and rabies) expressing NiV F or NiV G (68–73), recombinant measles virus vector expressing NiV G (74) – all of which have shown protection from NiV challenge in hamsters, pigs, ferrets and/or AGMs. The third, utilizes human monoclonal antibodies for passive prophylaxis – including m102.4 (directed against HeV G) (75–78) and 5B3 (directed against NiV F) (44, 79). Antibodies specific for G or F glycoproteins can neutralize virus but G appears to be the dominant neutralizing target (39, 65, 66). Based on experience with related paramyxoviruses and pneumoviruses, both the NiV F and G proteins are considered relevant protective antigens and targets for vaccine-elicited neutralizing antibodies.

The objectives for this study were to develop NiV vaccines by stabilizing the fusion protein in its prefusion conformation, designing multimeric G immunogens, and combining pre-F and G antigens to produce an immunogen that targets both surface glycoproteins. Here, we demonstrate that structure-based design can be utilized to develop highly immunogenic NiV vaccines and that these vaccine designs are transferable to related henipaviruses.

## Materials and Methods

### Structure-Based Design of Prefusion NiV F Glycoprotein Trimers

**FIGURE 1:**
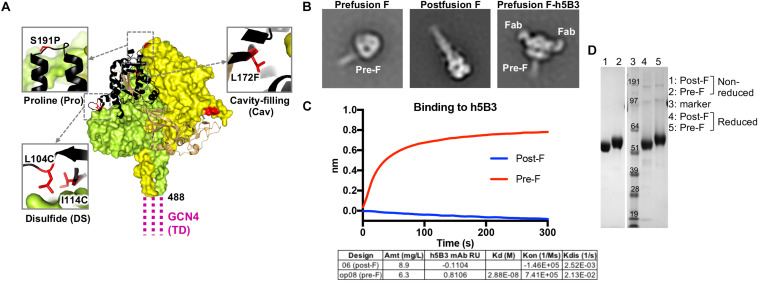
Structure-based design of a stabilized prefusion NiV F trimer. **(A)** Structure of prefusion NiV F glycoprotein trimer (PDB ID 5EVM) in green, orange and sand, and with residues that undergo >5 Å conformational change to transition to the postfusion conformation shown in black. GCN4 trimerization (TD) motif is shown in magenta, linked to NiV F residue 488. Zoom insets highlight mutated residues (red) to stabilize the prefusion F structure including L104C-I114C disulfide bond, a S191P helix-breaking proline substitution and a L172F cavity-filling mutation. **(B)** Two-dimensional class averages of the prefusion F trimer (left), postfusion F trimer (middle) and prefusion F trimer bound to h5B3 Fab (right) obtained by negative-stain electron microscopy (EM). **(C)** Binding kinetics were measured using a fortéBio Octet Red384 instrument. Table summarizing binding affinities of F designs to monoclonal antibody h5B3. **(D)** Non-reduced and reduced SDS-PAGE analysis of prefusion and postfusion NiV F glycoproteins.

**FIGURE 2:**
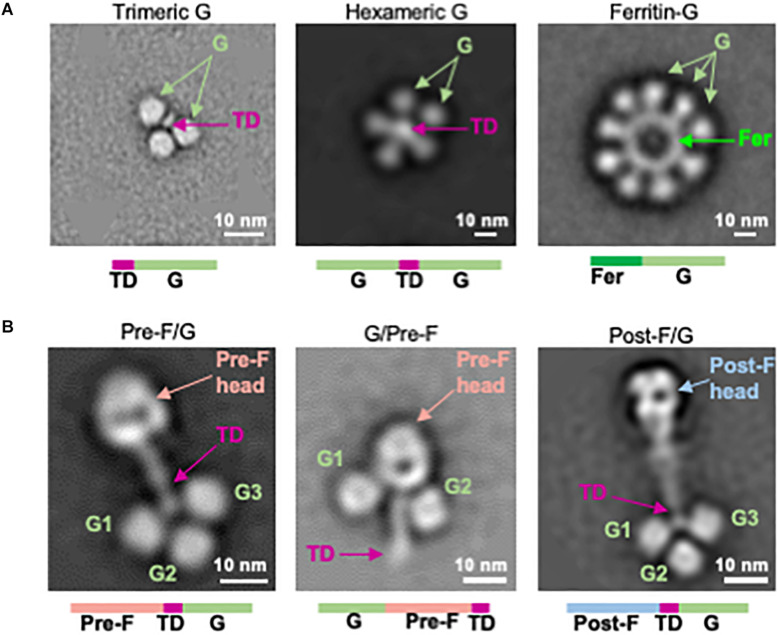
Structure-based design of NiV G immunogens and NiV F/G chimeric immunogens. **(A)** Two-dimensional class averages of NiV G head domain multimer designs (left: trimeric G, center: hexameric G, right: ferritin-G) obtained by negative-stain EM. **(B)** Negative-stain EM analysis of NiV F/G chimeras, showing pre-F/G (left), G/pre-F (center), and post-F/G (right). TD, trimerization domain.

**FIGURE 3:**
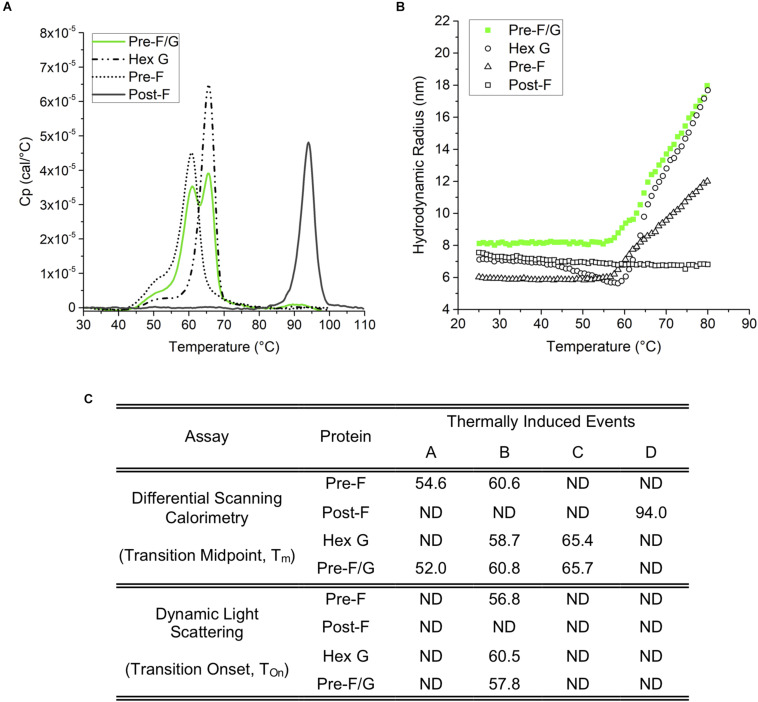
Thermodynamic and colloidal stability assessment of Pre-F, Post-F, Hex G, and Pre-F/G constructs. **(A)** Differential scanning calorimetry; based on the T_*m*_ (transition midpoint), the thermodynamic stability of the individual domains can be ranked Post-F>> Hex G > Pre-F, and Pre-F/G. **(B)** Dynamic light scattering indicates a similar degree of colloidal stability for Hex G, Pre-F and Pre-F/G, while Post-F is extremely colloidally stable, as indicated by lack of change in size within the temperature range of the experiment. **(C)** Table summarizing the conformational transitions **(A)** and colloidal stability **(B)** of Pre-F, Post-F, Hex G, and Pre-F/G.

Using the model from the NiV prefusion ectodomain glycoprotein crystal structure (PDB ID 5EVM), we designed 46 disulfide bonds, 16 cavity-filling mutations, 16 helix-disrupting mutations, 8 deletion/linker mutations, 5 glycine swap mutations and 16 combinations of disulfides, cavity-filling and/or helix-breaking mutations in the NiV Malaysia fusion protein sequence (GenBank accession number AAK50544.1; Supplementary Table S1, summary of key designs). By co-expression of the h5B3 Fab (NiV F-specific antigen binding fragment composed of one constant and one variable domain of each of the heavy and light chain and engineered with a premature stop codon following CH1) with the NiV F ectodomain glycoprotein with a C-terminal GCN4 coiled coil trimerization domain, we isolated antibody-F complexes which showed clear prefusion conformation for the F trimer (Figure 1B, right) with the h5B3 Fab binding in a 3:1 ratio (Fab:trimer) near an epitope similar to the RSV F site V. Using the NiV prefusion F-specific antibody h5B3 and an anti-Strep Tag II antibody (IBA), the designs were assessed for their ability to specifically bind the prefusion-specific antibody and express at high yield (Figure 1C and Supplementary Figure S1B) relative to the NiV F wild-type protein (expresses at <0.1 mg/L). F constructs yielding high binding titers to the prefusion-specific antibody were expressed and biophysical, structural and antigenic characteristics determined to confirm the prefusion conformation (Figures 1, 3). Using negative-stain EM as a readout for conformation, prefusion-stabilized NiV F ectodomain glycoprotein trimers containing various designs displayed almost 100% of the prefusion conformation (Figure 1B and Supplementary Figure S1C). Chimeric F and G containing immunogens were then made by adding linkers to expressing and characterized designs of pre F, post F, and G soluble ectodomain headgroup residues 172–602 or 177–602 (GenBank accession number AAK50545.1). These designs were evaluated for homogeneity via gel filtration, antigenic reactivity and negative-stain EM (Figures 2, 3 and Supplementary Figure S2).

### Protein Expression and Purification

NiV F, G, or F/G glycoproteins were expressed by transfection in 293 FreeStyle (293F) cells (Thermo Fisher Scientific, Waltham, MA, United States) using Turbo293 transfection reagent (SPEED BioSystem, Gaithersburg, MD, United States) according to the manufacturer’s protocol. Transfected cells were incubated in shaker incubators at 120 rpm, 37°C, 9% CO_2_ overnight. On the second day, one tenth culture volume of Cell Booster medium (ABI Scientific, Sterling, VA, United States) was added to each flask of transfected cells and cell cultures were incubated at 120 rpm, 37°C, 9% CO_2_ for an additional 4 days. Five days post-transfection, cell culture supernatants were harvested and proteins were purified from the supernatants using tandem Ni^2+^ (Roche) and Strep-Tactin (IBA) affinity purification. The C-terminal purification tags were removed by thrombin digestion at room temperature overnight and proteins were further purified by SEC in a Superdex 200 column (GE) in 1x phosphate-buffered saline (PBS).

### Negative-Stain Electron Microscopy

Proteins were diluted to approximately 0.01–0.02 mg/mL with 10 mM HEPES, pH 7.0, 150 mM NaCl, adsorbed to a freshly glow-discharged carbon-coated grid, washed with the same buffer, and stained with 0.7% uranyl formate. Datasets were collected at a magnification of 100,000 using SerialEM (80) on an FEI Tecnai T20 microscope equipped with a 2k x 2k Eagle CCD camera and operated at 200 kV. The nominal magnification was 100,000 and the pixel size was 0.22 nm. Particles were selected from micrographs automatically using in-house written software (YT, unpublished), followed by manual correction using EMAN2 (81), when necessary. Reference-free 2D classifications were performed with Relion 1.4 (82). Fractions of prefusion and postfusion molecules were determined by calculating the numbers of particles that contributed to prefusion and postfusion classes.

### Generation of Monoclonal Antibodies

Coding sequences for the heavy and light chains of the NiV F-specific antibody, h5B3 or HeV G-specific antibody, m102.4, containing the human consensus sequence for IgG1 (heavy chain) and kappa (light chain), were synthesized by GeneArt (Thermo-Fisher Scientific, St. Louis, MO, United States). Both were cloned into the VRC8400 vector using *Xba*I/*Sac*II (heavy chain) or *Xba*I/*Bam*HI (light chain). h5B3 heavy and light chain sequences were acquired from Patent Application US2016/0347827 A1 (83). m102.4 heavy and l ight chain sequences were acquired from Patent US7988971 B2 (84). Expi293 suspension cells (50 mL at 1.5-3e6 cells/mL) were transfected with 50 μg of heavy chain and 50 μg of light chain using Expifectamine transfection reagent according to manufacturer’s instructions. Five days post-transfection, supernatant was collected and centrifuged, protease inhibitor were added to clarified supernatant and purified using protein A-agarose resin. Bound antibody is eluted with IgG elution buffer into 1/10th volume of 1M Tris–HCl (pH 8.0).

### Antigenic Screening of NiV F, G, F/G Chimera Immunogens

Initial assessment of all constructs were performed using a 96-well microplate format for high throughput expression followed by an ELISA-based antigenic evaluation as described previously (48). Briefly, 24 h prior to transfection HEK 293T cells (Thermo Fisher Scientific, Waltham, MA, United States) were seeded in each well of a 96-well microplate at a density of 2.5 × 10^5^ cells/mL in expression medium (high glucose DMEM supplemented with 10% fetal bovine serum and 1% Pen/Strep, 1% GlutaMax), and incubated at 37°C, 5% CO_2_ for 20 h. Plasmid DNA and TrueFect-Max (United BioSystems, College Park, MD, United States) were mixed and added to the growing cells, and the 96-well plate incubated at 37°C, 5% CO_2_. One day post transfection, enriched medium (high glucose DMEM plus 25% ultra-low IgG fetal bovine serum, 2x non-essential amino acids, 1x glutamine) was added to each well, and the 96-well plate was returned to the incubator for continuous culture. Five days post-transfection, supernatants with the expressed NiV F, NiV G, or NiV F/G variants were harvested and tested by ELISA for binding to h5B3 and m102.4 antibodies using Ni^2+^-NTA microplates.

### Sample Preparation for Dynamic Light Scattering (DLS) and Differential Scanning Calorimetry (DSC)

Sample were diluted in PBS to a concentration of 0.5 mg/mL for DSC and 1 mg/mL for DLS. DLS samples were filtered with a 0.1 μm, 10 mm diameter PES syringe filter.

### Thermal Unfolding Transition by Dynamic Light Scattering (DLS)

Samples were evaluated by Dynamic Light Scattering (DLS) when subjected to a thermal ramp using the DynaPro Plate Reader II (Wyatt Technology, Santa Barbara, CA, United States). Samples were assayed (*n* = 3) in a 384 well plate; each sample well was filled with 30 μL sample and topped with 10 μL high-purity paraffin oil (Sigma-Aldrich, St. Louis, MO, United States) to prevent evaporation. The wells surrounding the samples were filled with paraffin oil to mitigate edge effects. Each datapoint was generated from 5 readings (5 s acquisition time) for each well during a continuous thermal ramp from 25°C to 80°C @ 0.12°C min^–1^. Particle data were reported for cumulant R_*h*_ values in the range of 2–5000 nm. The thermal transition onset (*T*_*onset*_) for each sample was determined using the onset function in Dynamics Software, version 7.8.0 (Wyatt Technology, Santa Barbara, CA, United States). Data was not viscosity corrected.

### Differential Scanning Calorimetry (DSC)

Differential Scanning Calorimetry (DSC) thermograms were acquired at 0.5 mg/mL sample concentration using a MicroCal VP-Capillary DSC (Malvern Panalytical, Westborough, PA, United States). Heat differential was monitored as the sample cell temperature was increased from 5°C to 100°C (110°C for the postfusion F protein) at a rate of 60°C/h. Thermograms were subjected to mathematical deconvolution using the MicroCal LLC DSC plug-in for Origin Software (ver. 7.0) to resolve underlying peaks and determine transition midpoints (T_*m*_). Buffer-subtraction and baseline correction were applied.

### Antigenic/Immunogenic Characterization of NiV F, G, F/G Chimeric Immunogens

A fortéBio Octet Red384 instrument was used to measure binding kinetics of NiV F, NiV G, or NiV F/G variants to an antibody recognizing prefusion F (h5B3) or HeV G (m102.4). The fortéBio Octet Red384 instrument obtains real-time kinetic binding data [reviewed in (85)]. All assays were performed with agitation set to 1,000 rpm in 1xPBS supplemented with 1% bovine serum albumin (BSA) to minimize non-specific interactions. The final volume for all solutions was 60 μL/well. Assays were performed at 30°C in tilted black 384-well plates (Geiger Bio-One). For protein binding to antibody – human Fc IgG sensor tips were used to capture either h5B3 or m102.4 antibodies (diluted to 20–30 μg/mL) onto the sensor tips, followed by association of the loaded antibody with NiV/HeV/CedPV protein variants (diluted to 20–30 μg/mL). For determination of F-specific or G-specific antibodies from sera collected from mice immunized with NiV protein variants – Ni^2+^-NTA, His1k or streptavidin sensor tips were used to capture NiV prefusion F or NiV monomeric G protein using His-streptavidin tag (diluted to 20–30 μg/mL), followed by association of the loaded protein with binding antibodies in sera (diluted 1:200). Typical capture levels for each loading step were between 1.4 and 1.5 nm, and variability within a row of eight tips did not exceed 0.1 nm for each of these steps. Biosensor tips were equilibrated for 60 s in 1xPBS + 1% BSA prior to loading antibody or protein variants. Biosensor tips were then equilibrated for 120 s in 1xPBS + 1% BSA prior to measuring association with protein variants or sera from mice immunized with NiV variants in solution for 600 s; protein or sera was then allowed to dissociate for 600 s. Parallel correction to subtract systematic baseline drift was carried out by subtracting the measurements recorded for a loaded sensor incubated in 1xPBS + 1% BSA. Data analysis and curve fitting were carried out using Octet software, version 9.0. Experimental data were fitted with the binding equations describing a 1:1 interaction. Global analysis of the data sets assuming reversible binding (full dissociation) were carried out using non-linear least-squares fitting allowing a single set of binding parameters to be obtained simultaneously for all of the concentrations used in each experiment.

### Animal Immunizations

All animal experiments were reviewed and approved by the Animal Care and Use Committee of the Vaccine Research Center, NIAID, NIH, and all animals were housed and cared for in accordance with local, state, federal and institute policies in an American Association for Accreditation of Laboratory Animal Care (AAALAC)-accredited facility at the NIH. Groups of 10 CB6F1/J mice were immunized twice at weeks 0 and 3 intramuscularly with 5 μg or 10 μg of recombinant NiV F glycoprotein trimer designs, multimeric forms of G or F/G chimeric designs combined with 100 μg aluminum hydroxide (alum). Serum was collected at week 2 and 5 following immunization. Week 5 sera was assessed for immunogenicity in biolayer interferometry studies and for neutralization in a pseudovirus neutralization assay *in vitro*.

### Generation of NiV Pseudovirus

To obtain VSVΔG-luciferase pseudotyped with NiV F_*WT*_ and NiV G proteins, BHK21 cells were first co-transfected with VRC8400 NiV F_*WT*_ and VRC8400 NIV G. Transfected cells showing extensive cell-to-cell fusion were infected with VSV-G complemented VSVΔG-luciferase at an MOI of 4, about 24 h post-transfection. At 1 h post-infection, input virus was removed, cells were washed with 1xPBS and DMEM with 10% FBS, 1% Pen/Strep, 1% GlutaMax was added to the cells. Medium/cells containing VSVΔG-luciferase pseudotyped with NiV F_*WT*_ and G was collected after 24 h and sonicated, before being clarified. Stock pseudovirus was confirmed to have incorporated both NiV F and NiV G by demonstrating h5B3 mAb and m102.4 mAb were able to neutralize pseudovirus infectivity individually in a luciferase assay.

### Immunogenic Characterization of NiV F, G, and F/G Chimeric Designs in Mice

A pseudovirus neutralization assay is used because NiV is classified as a BSL 4 pathogen. Neutralizing antibody titers were determined using a microneutralization assay using VSVΔG-luciferase expressing NiV F_*WT*_ and NiV G in Vero E6 cells as previously described (86). NiV F/G VSVΔG-luciferase pseudovirus was first incubated with anti-VSV G monoclonal antibody (8G5) for 15 min to neutralize any trace infection due to residual VSV G that may have been incorporated into the particles pseudotyped with NiV F_*WT*_ and G proteins. Serum samples were heat-inactivated at 55°C for 30 min. Serum samples or pooled serum samples from each immunization group were serially diluted in DMEM with 10% FBS, 1% Pen/Strep, 1% GlutaMax and mixed with equal volume of pseudotyped particles with anti-VSV G antibody, incubated for 30 min at room temperature before addition to Vero E6 cells. After 24 h, medium was removed by aspiration, plates were washed with 300 μL 1xPBS/well. Cell lysis and detection of firefly luciferase were performed according to the protocol recommended by the manufacturer (Promega Inc.). Briefly, firefly luciferase assay lysis buffer was thawed to room temperature, diluted 1:5 with ddH_2_O and 20 μL was added to each well. Plates were placed on an orbital shaker for 20–30 min. Following lysis, 50 μL of luciferase assay reagent was added to each well and read at 570 nm on the SpectraMax L luminometer (Molecular Devices). The 80% inhibitory concentration (IC_80_) was calculated by curve fitting and non-linear regression of average RLU number in triplicate wells using GraphPad Prism. The histologic scoring data were analyzed by one-way analysis of variance (ANOVA), and Tukey’s multiple-comparison test was used to evaluate differences between vaccine groups.

## Results

### Structure-Based Design of Prefusion-Stabilized NiV F

We analyzed the NiV prefusion F glycoprotein structure (PDB ID 5EVM) (47) and designed approximately 150 variants intended to stabilize the NiV F glycoprotein trimer in its native prefusion conformation. The mutations were made in regions of F predicted to undergo conformational change to the postfusion form and include disulfide bonds, cavity-filling side chains, helix disrupting mutations, glycine turns, fusion peptide deletion mutations and the use of a C-terminal GCN4 trimerization motif. The F ectodomain glycoprotein designs were assessed for expression yield relative to the NiV F wild-type protein (expression yield is <0.1 mg/L), molecular size and homogeneity via size-exclusion chromatography profiles, antigenic recognition by the humanized prefusion NiV F-specific monoclonal antibody, h5B3 (44, 79) and prefusion versus postfusion conformation via negative-stain electron microscopy (Figure 1). A series of second-generation designs were produced by combining the first-generation designs with the highest protein expression, percentage of prefusion conformation by negative-stain EM, and binding to h5B3. Several lead pre-F candidates resulted in approximately 50-fold increase in protein expression compared to wild-type NiV F, often a correlate for protein stability (Supplementary Figure S1B). Some designs showed monodispersed chromatograms on size-exclusion purification at the expected trimeric molecular weight (Supplementary Figures S1A,D). Another single-chain uncleaved design, NiV06 (post-F) with a GGS linker replacing a deletion between N99 and G117, was homogenously of postfusion F conformation (Figure 1B, center).

Prefusion NiV F ectodomain glycoprotein designs and complexes with h5B3 Fab were further analyzed by negative-stain EM to determine their homogeneity and the ratio of pre-F/post-F conformations (Figure 1B and Supplementary Figure S1C). The h5B3 antibody did not bind this postfusion F design demonstrating that h5B3 is prefusion F-specific. The 2D negative-stain EM images of the h5B3 Fab bound to NiVop08 (pre-F) revealed a binding position whereby the antibody attaches to a region of the prefusion F predicted to undergo substantial conformational rearrangement during transition to post-F, thereby explaining the pre-F specificity of this antibody (Figure 1B, right). h5B3 was originally isolated from mice immunized with NiV soluble F variants and described as a conformationally dependent antibody (44). The interaction of h5B3 bound to NiV prefusion F has been further characterized (79). NiVop08 was ultimately selected as the lead prefusion-stabilized F design due to its favorable biophysical and antigenic characteristics. This variant contains the mutations I114C-L104C, L172F, and S191P and a C-terminal GCN4 trimerization motif following residue 488 (Figure 1A). After affinity column and size-exclusion chromatography purification, NiVop08 F glycoprotein trimer bound h5B3 with a K_*d*_ affinity of 2.9 × 10^–8^M (Supplementary Figures S1A,B) and 100% of the F protein particles were in the prefusion conformation based on negative-stain EM (Figure 1B, left, and Supplementary Figure S1C).

### Design of Multimeric Forms of NiV G

Next, we designed monomeric and oligomeric NiV G vaccine candidates. The globular soluble head domain residues 172-602 of the NiV attachment protein (G) were expressed with a C-terminal thrombin-cleavable purification tag with yields of 3.8 mg/L in 293F cells (Supplementary Figures S2C,D). The same region of NiV G was coupled to an N-terminal foldon domain yielding 1.8 mg/L of homogeneous G trimer (Figure 2A, left, and Supplementary Figures S2A,C–E). A hexameric NiV G (hex G) was designed by linking two G ectodomain headgroups by a foldon trimerization domain via short glycine linkers (Figure 2A, center). The yield was 2.3 mg/L (Supplementary Figures S2A,C–E). A NiV G ferritin 24-mer nanoparticle was formed by linking the N-terminus of the NiV G ectodomain residues 172–602 to the C-terminus of ferritin via a G_3_SG_2_ linker (Figure 2A, right, and Supplementary Figure S2A). All G multimer designs demonstrated antigenic reactivity with G-specific m102.4 antibody (Supplementary Figure S2C) with differences in binding to m102.4 antibody attributed to the number of Gs present in the design. None of the G multimer designs bound to the humanized prefusion NiV F-specific monoclonal antibody, h5B3 (Supplementary Figure S2D). A soluble version of the native tetrameric form (stalk G) of NiV composed of residues 72–602, similar to the Equivac HeV sG vaccine was constructed. The NiV stalk G design expressed to comparable levels as hexameric G with similar gel filtration profiles (Supplementary Figure S2A).

### Design of NiV Pre-F/G Chimeras

NiV F and NiV G were linked directly to form chimeric immunogens comprising the major targets of NiV neutralizing antibodies. The prefusion-stabilized F design NiVop08 (pre-F) was linked at the C-terminus to the trimerization motif which was linked to the N-terminus of NiV G residues 177–602 via a GSG_5_ linker to form a covalent single-chain pre-F/G design (Figure 2B, left). A G/pre-F design was formed by linking NiV G residues 177–602 at the N-terminus of NiVop08 (pre-F) via a G_4_SG_4_ linker (Figure 2B, center). Similarly, a post-F/G design was made by linking the postfusion-stabilized F NiV06 (post-F) with a C-terminal trimerization motif to NiV G residues 177–602 via a GSG_5_ linker (Figure 2B, right). These designs were analyzed by SDS-PAGE (Supplementary Figure S2F), gel filtration and binding to m102.4 and h5B3 antibodies which confirmed the anticipated antigenic properties and monodispersity of these protein designs (Supplementary Figures S2B–D).

The pre-F/G, post-F/G, and G/pre-F negative-stain EM analyses suggested that the sites of neutralization were solvent exposed. This was confirmed by antigenic reactivity with pre-F-specific h5B3 or G-specific m102.4 antibodies (Supplementary Figures S2C,D). The G/pre-F chimeric protein had a comparatively more compact structure, with the G monomers located at the base of the prefusion NiV F head adjacent to the stem domain. This arrangement was similarly reactive to both antibodies m102.4 and h5B3 as other pre-F and G designs, with comparable affinity constants (Supplementary Figures S2C,D), indicating the m102.4 and h5B3 neutralization epitopes are accessible. Pre-F/G is the lead chimeric protein design based on protein expression levels, efficiency of purification, and F- and G-specific antibody binding responses. All NiV F, G, and F/G designs are summarized in Supplementary Table S1.

### Biophysical Characterization of NiV Immunogens

An evaluation of the thermodynamic conformation and colloidal stability of pre-F, post-F, hex G, and the pre-F/G chimera was performed. Differential scanning calorimetry thermograms (Figure 3) identified onset of presumed conformational transitions at temperatures of ∼45°C for pre-F, hex G, and pre-F/G proteins. The primary transition midpoints (T_*m*_) of the pre-F and hex G proteins (∼60 and ∼65°C, respectively) were observed in the pre-F/G chimera, suggesting minimal long-range interaction between the protein domains within the chimera (Figures 3A,C). The post-F protein possessed an extremely high intrinsic stability, remaining stable well above 80°C. Colloidal stability of the four proteins, as assessed by dynamic light scattering analysis, showed similar trends in T_*onset*_ to the DSC thermograms, suggesting that the main structural transition of the pre-F, hex G and pre-F/G proteins coincided with a heat-induced aggregation event (Figures 3B,C). No aggregation of the post-F protein was detected, although instrument limitations prevented analysis above 80°C. The biophysical characterization of our lead NiV protein designs indicate they are stable and well-behaved proteins.

### Immunogenicity of NiV F, NiV G, and NiV F/G Chimeras in Mice

**FIGURE 4:**
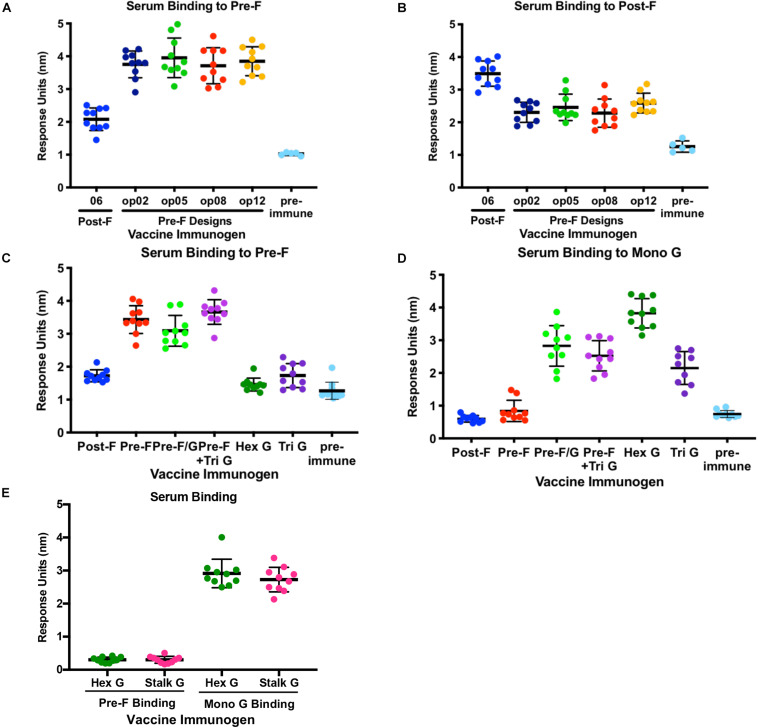
Immunogenicity of NiV Pre-F stabilized immunogens, multimeric forms of G and Pre-F/G chimeric immunogens. **(A,B)** Recognition of pre-F **(A)** or post-F **(B)** NiV F proteins by sera from mice immunized twice with NiV F designs or unimmunized. **(C,D)** Recognition of pre-F NiV F **(C)** or mono G **(D)** protein by sera from mice immunized twice with NiV F, NiV G multimers or NiV F/G chimeric designs or unimmunized. **(E)** Recognition of pre-F NiV F or mono G proteins by sera from mice immunized twice with NiV Hex G or NiV Stalk G. Binding kinetics were measured using a fortéBio Octet Red384 instrument. Line represents mean of all animals in each group ± standard deviation (using GraphPad Prism).

To understand how conformation of the NiV fusion protein, multimeric forms of G or chimeric F/G proteins affect immunogenicity, groups of 10 CBJF1/J mice were immunized with 5 μg or 10 μg/dose of purified pre-F and post-F trimeric glycoproteins, multimeric forms of NiV G or pre-F/G chimeras with 100 μg aluminum hydroxide (alum) at weeks 0 and 3 (Supplementary Figure S3A). At 2 weeks post-second immunization, sera were analyzed for binding to pre-F, post-F and/or monomeric G antigens using biolayer interferometry for qualitative, rather than quantitative, comparison of the elicited immune response. All animals had robust F-specific antibody responses (Figures 4A,B and Supplementary Figures S3B,C). Pre-F-immunized mouse serum displayed high levels of antibody binding responses to pre-F antigen (NiVop08) while showing comparatively lower antibody binding responses to post-F antigen (NiV06) (Figures 4A,B and Supplementary Figures S3B,C). Mice immunized with all of the pre-F designs showed similar levels of binding to pre-F and post-F. Conversely, postfusion-immunized mouse serum had higher antibody binding responses to post-F antigen and comparatively lower antibody binding responses to pre-F antigen (Figures 4A,B and Supplementary Figures S3B,C). These data suggest that there are a significant number of antigenic sites that are not shared between the pre-F and post-F molecules and that most antibodies induced by F immunogens are conformation-specific.

All animals immunized with multimeric forms of G elicited G-specific antibody responses only (Figures 4C,D and Supplementary Figures S3D,E) while mice immunized with pre-F/G chimeras or pre-F + G generated antibody responses directed against both prefusion F and monomeric G proteins (Figures 4C,D and Supplementary Figures S3D,E). The hexameric form of NiV G elicited significantly more G-specific antibodies than monomeric or trimeric G and similar antibody responses to the native tetrameric (dimer of homodimers) NiV G (stalk G) and the ferritin-G designs (Figure 4E and Supplementary Figure S3E). The pre-F/G chimera elicited more F-specific antibodies than either post-F/G chimeras or the G/pre-F chimera, likely due to the absence of neutralization-sensitive epitopes on post-F and poor access to antigen sites on G/pre-F where protein is packed at the base of the F protein head. All F/G chimeras had G-specific antibody responses. The pre-F/G chimera and pre-F + trimeric G immunized groups elicited similar F- and G-specific antibody responses.

**FIGURE 5:**
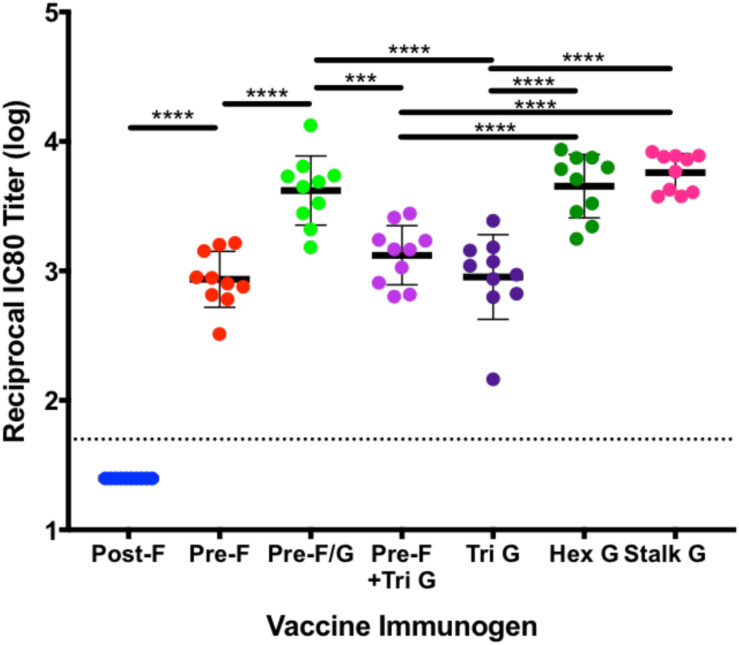
Neutralization of NiVF/G VSVΔG-luciferase pseudovirus by sera from mice immunized with NiV stabilized pre-F, multimeric forms of G and F/G chimeric immunogens. VSVΔG-luciferase pseudovirus (expresses both NiV F_*WT*_ and NiV G on surface) neutralization assays were performed on individual mouse sera collected at week 5. The log_10_ reciprocal IC_80_ titer for each sample was calculated by curve fitting and non-linear regression using GraphPad Prism. *P*-values were calculated using one-way ANOVA with Tukey’s multiple comparisons test (***p < 0.001, ****p < 0.0001). Line represents mean of log_10_ reciprocal IC_80_ titer ± standard deviation (using GraphPad Prism).

To assess the ability of recombinant NiV prefusion F trimer designs, multimeric forms of G or F/G chimeric immunogens to elicit neutralizing antibodies, serum was evaluated in the NiV F/G VSVΔG-luciferase pseudovirus system, similar to what was described previously (86, 87). Individual mouse serum was serially diluted for selected groups (Figure 5) or pooled from 10 animals in each group for all selected prefusion F protein designs, multimeric forms of G and F/G chimeric designs (Supplementary Table S2). No detectable neutralizing activity was observed in sera from post-F-immunized mice while sera from pre-F-immunized mice (05, 09, op02, op05, op06, op08, and op13) neutralized NiV F/G VSVΔG-luciferase pseudovirus with a reciprocal IC_80_ titer of >1000 (Figure 5 and Supplementary Table S2). Immunogens that elicited robust pre-F-specific antibody binding responses also elicited robust neutralizing antibody responses (Figures 4A, 5). The hex G elicited higher neutralizing activity (reciprocal IC_80_ titer >3400) relative to monomeric (reciprocal IC_80_ titer <350) or other multimeric G immunogens (reciprocal IC_80_ titer <2200) (Supplementary Table S2). The pre-F/G chimeric immunogen elicited a potent neutralizing antibody response, comparable to the hexameric or stalk (native) G, achieving a reciprocal IC_80_ titer of >6700 (Figure 5 and Supplementary Table S2). The neutralizing activity elicited by the pre-F/G chimera was statistically higher than those elicited by pre-F, tri G, or pre-F + tri G (Figure 5).

### Application of NiV Immunogen Designs to Phylogenetically Related Paramyxovirus Glycoproteins

The similarity of the NIV and HeV genomes with high amino acid sequence homology between the F (88%) and G (83%) proteins (43, 88) and identification of highly potent cross-reactive neutralizing antibodies (44, 79, 89) suggested NiV pre-F stabilizing designs may be transferred into HeV F constructs to yield a stabilized prefusion HeV F glycoprotein. The lack of homology between the F (43%) and G (30%) proteins (88) of NiV with Cedar virus suggested that application of the NiV immunogen designs would have a higher risk of failure. Antibodies to Cedar virus cross-react with but do not cross-neutralize either NiV or HeV viruses (88).

**FIGURE 6:**
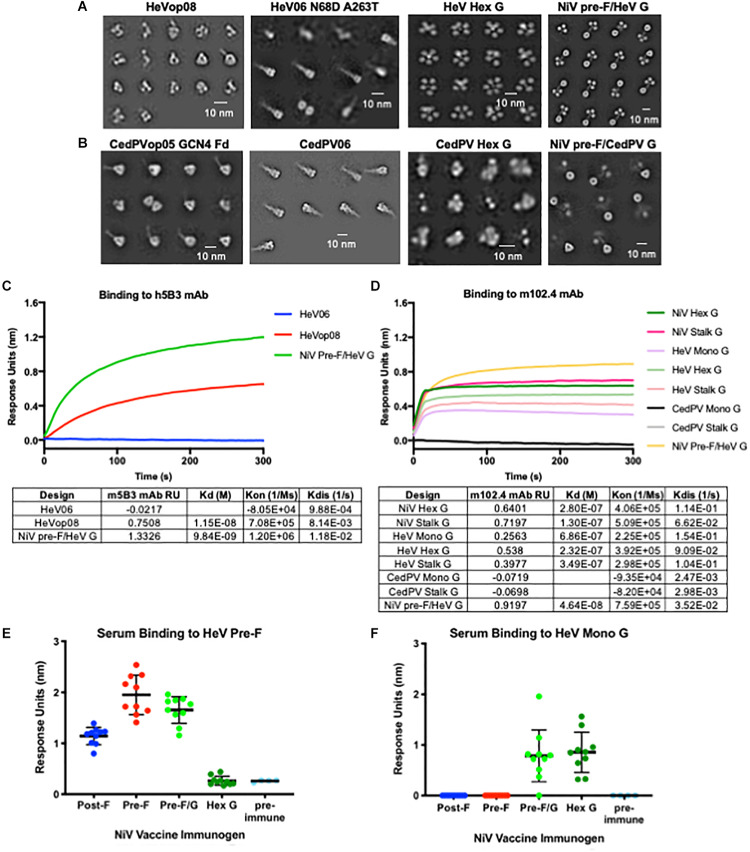
Application of NiV antigen designs to phylogenetically related Henipaviruses. **(A,B)** Two-dimensional negative-stain EM class averages of NiV designs applied to Hendra **(A)** and Cedar **(B)** viruses. **(C)** Binding kinetics of HeV F pre-F, post-F, and NiV pre-F/HeV G chimeric immunogen binding to h5B3, NiV pre-F-specific antibody. **(D)** Binding kinetics of multimeric forms of NiV G, HeV G, CedPV G and NiV pre-F/HeV G chimeric immunogen binding to m102.4, HeV G-specific antibody. **(E,F)** Recognition of HeV pre-F **(E)** or HeV Mono G **(F)** proteins by sera from mice immunized twice with NiV F, G or F/G immunogens. Binding kinetics were measured using a fortéBio Octet Red384 instrument. Line represents mean of all animals in each group ± standard deviation (using GraphPad Prism).

We transferred several NiV stabilizing pre-F mutations (05, op02, op05, op08, and op12) to both HeV F and Cedar virus F, and applied the NiV post-F mutations and hex G designs to the homologous HeV and Cedar virus proteins (Figures 6A,B). The HeV pre-F mutation corresponding to NiVop08 stabilized F in the prefusion conformation, but protein expression levels from transfected cells were modestly improved relative to wild-type HeV F. The NiV pre-F design that worked best to stabilize the Cedar virus fusion protein in its prefusion conformation corresponded to NiVop05, with two trimerization domains added (GCN4 and foldon). This design did not improve protein expression above wild-type levels in transfected cells. The NiV post-F design (06) was transferable to both HeV and Cedar virus post-F constructs, but again did not significantly increase protein yield over wild-type sequences. In the HeV design, two additional mutations present in some isolated sequences (N68D and A263T) were added (52, 90). Antigenic characterization of the HeV F designs showed that the pre-F design, but not post-F design, bound to h5B3, the NIV prefusion F-specific antibody (Figure 6C) suggesting that at least some of the protein has been stabilized in the prefusion conformation. The hex G design transferred to HeV G with protein expression levels comparable to NiV hex G, but not to Cedar virus G, which was disordered based on negative-stain EM (Figures 6A,B). HeV G designs (monomeric, hexameric and native tetrameric stalk) bound to the HeV G-specific antibody, m102.4 (Figure 6D) although not as well as NiV G designs which is consistent with previous findings that NiV G binds m102.4 better than HeV G (75). Additionally, we combined our NiV pre-F design with either HeV G or CedPV G (Figures 6A,B; far right). The NiV pre-F/HeV G chimera expressed well and showed a stable protein structure on negative-stain EM; however, the NiV pre-F/CedPV G chimera did not express well and the G domains were not fully resolved by negative-stain EM. We were able to transfer the NiV stabilized pre-F designs and hexameric G designs directly to HeV to stabilize protein structure and to modestly increase protein expression yields. However, we were unable to reliably transfer NiV stabilized pre-F designs or hexameric G designs directly to Cedar virus, suggesting additional empirical refinement is needed for production of vaccine antigens. These findings underscore the importance of having access to atomic level protein structure information to guide the immunogen design process.

### Hendra Virus Cross-Reactive Antibody Responses

Previous studies have shown NiV and HeV antisera cross-neutralize, with each serum being slightly less effective against the heterotypic virus (39, 91). NiV and HeV glycoproteins functionally complement one another in mediating membrane fusion with wild-type efficiency (52, 92). Therefore, we asked whether antibodies elicited from mice immunized with NiV antigens could bind HeV antigens. Sera collected from mice immunized with the lead vaccine candidates were analyzed for binding to HeV prefusion F-stabilized antigen and HeV G monomeric antigen using biolayer interferometry (Figures 6E,F). All animals immunized with pre-F, post-F, or pre-F/G elicited F-specific antibody responses that cross-react with HeV pre-F. NiV pre-F-immunized animal sera displayed higher antibody binding responses to the HeV pre-F antigen than sera from NiV post-F-immunized animals, similar to what was seen with NiV pre-F (Figure 3A). No F antibody binding response was elicited from animals immunized with hex G. Animals immunized with pre-F/G or hex G elicited G-specific antibody responses directed against HeV monomeric G. No antibody binding responses were elicited from animals immunized with pre-F or post-F. Cross-reactivity of NiV sera against HeV pre-F and/or HeV G suggests that NiV immunogens may be sufficient to protect against HeV in the case of a pandemic given their amino acid homology.

## Discussion

Protein structure is a powerful tool when applied to design and development of vaccine immunogens (93, 94). We used knowledge from previous immunogen design efforts for RSV and paramyxoviruses (45, 48), including NiV (44), to stabilize NiV F protein in its prefusion conformation. Additionally, we constructed multimeric forms of NiV G and covalently linked the stabilized pre-F and G to form a chimeric protein. These NiV designs elicited potent neutralizing antibodies that were cross-reactive with HeV proteins. The lead vaccine designs were transferred to phylogenetically related henipaviruses and may represent a generalizable solution for paramyxovirus vaccine immunogen development.

We found that prefusion-stabilized NiV F induced more potent neutralizing activity than postfusion F, supporting the importance of stabilizing the prefusion conformation to increase immunogenicity, as previously observed with RSV F and PIV1-4 F (45, 48, 50). Solving the crystal structure of the RSV F protein in its prefusion conformation led to the discovery of highly neutralization-sensitive epitopes at the apex of pre-F that were absent in the rearranged post-F structure. The antibodies targeting RSV pre-F specific antigenic sites Ø and V are particularly potent and have significantly greater neutralizing activity than antibodies directed against shared antigenic sites (II, III, and IV) (95, 96). The stabilized pre-F protein for human metapneumovirus (hMPV), a Pneumovirus closely related to RSV, showed similar immunogenicity to the post-F variant with structural data revealing the majority of hMPV neutralization-sensitive epitopes were on the shared surfaces of pre-F and post-F (97). While the reported hMPV pre-F was not a superior immunogen to post-F, it was equally potent for induction of neutralizing activity. Stabilized PIV3 pre-F induced neutralizing antibody titers 200- to 500-fold higher than the postfusion conformation in mice. The PIV1, PIV2, and PIV4 stabilized prefusion F proteins elicited neutralizing antibody titers 2- to 20-fold higher than corresponding postfusion immunogens (45). Stabilizing the prefusion conformation of other class I fusion proteins like the spike glycoprotein of coronaviruses has also resulted in more potent immunogens (98, 99). Results from biolayer interferometry indicate the stabilized prefusion F immunogen has some shared epitopes with the postfusion version. However, the NiV post-F failed to elicit significant neutralizing activity in mice suggesting that these shared epitopes are not targets for neutralizing antibodies. Therefore, the pre-F conformation would need to be maintained for a NiV vaccine to elicit effective F-specific neutralizing antibody responses. Previous research has shown similar findings (44, 79). Briefly, a soluble NiV F trimer, designed by deleting the transmembrane and cytoplasmic tail domains and adding a trimeric coiled-coil (GCN4) domain, was found to be in a prefusion conformation based on single-particle EM analysis and characterization with three NiV F monoclonal antibodies [including 5B3, a prefusion F-specific antibody; (79)] although this soluble F trimer could be triggered to the postfusion form with heat or trypsin-treatment. A postfusion form was generated by deleting the fusion peptide yielding a distinct elongated structure by EM (postfusion form) and did not bind to the NiV 5B3 conformation-dependent antibody. Additionally, only the soluble NiV F protein in its prefusion conformation was able to elicit neutralizing antibodies in mice that were cross-reactive with HeV (44).

The most advanced henipavirus vaccine in development to date is focused on the native G immunogen from HeV (57, 60, 61, 65, 67, 71–73, 100, 101). A HeV soluble native G tetramer is an effective immunogen and licensed for use in horses in Australia (58, 62, 64). This HeV vaccine has also been evaluated for protection against NiV virus and has been shown to induce cross-protective antibody responses in ferret challenge studies (59). In the current study, multimeric forms of NiV G elicited higher neutralizing antibody titers compared to monomeric G. Multimeric G designs evaluated here utilized a trimerization domain to form G trimers or hexamers or self-assembling ferritin nanoparticles that display 24 G monomers and were compared to the soluble native tetrameric form of NiV G. These designs all formed well-structured proteins by negative-stain EM. We did not characterize G hexamers composed of three NiV Gs and three HeV Gs or other combinations, but the multimer platforms (either hexamer or ferritin) allow for combinations of two or more different henipavirus G proteins, thereby expanding the potential breadth of vaccine-induced protection if needed. While G alone may be sufficient as a vaccine antigen, having both F and G antigens represented in a candidate vaccine theoretically increases the number of neutralization epitopes targeted to produce a broader multivalent polyclonal antibody response that would be more difficult to escape.

The pre-F/G chimera elicited higher neutralizing antibody responses than either of its component parts: pre-F or trimeric G. The pre-F/G chimeric protein designs represent a novel structurally designed immunogen, presenting epitopes of both surface glycoproteins required for entry. Chimeric antigen designs have been previously evaluated by linking the fusion protein (F) with the attachment protein (G or HN) (102–105). One study reported an RSV FG chimera containing the signal sequence and ectodomain of RSV F (residues 1–489) linked to the extracellular region of RSV G (residues 9–279), expressed in insect cells using a baculovirus vector that induced protective neutralizing activity in cotton rats (102, 105). Garg et al., expressed truncated, secreted forms of PIV3 F and HN proteins, individually and as a chimeric FHN protein (F protein residues 23–466; HN protein residues 87–572; linked via a glycine-serine linker) (103). The chimeric FHN protein was more immunogenic than the combination of F and HN proteins and intramuscular immunization with FHN/TriAdj elicited complete protection from PIV3 challenge in cotton rats and hamsters (103). As noted for the RSV FG, the PIV3 truncated fusion protein was most likely in a postfusion conformation and possibly monomeric instead of trimeric implying that some of the most potent neutralizing components of the fusion protein were not intact or present. We were able to reference atomic level structural information when designing our NiV pre-F/G chimera to make mutations that stabilized the fusion protein in the preferred prefusion trimeric conformation using disulfide and cavity-filling mutations while retaining a GCN4 trimerization motif at the C-terminus. The improved immune response with the chimeric pre-F/G proteins compared to the mixture of pre-F and trimeric G proteins (Figure 5 and Supplementary Table S2) may be due to improved intramolecular CD4 T cell help since this improvement was also seen with both RSV FG and PIV3 FHN chimeras (102, 103).

The chimeric protein design approach allows for the inclusion of glycoproteins from more than one henipavirus. For example, combining NiV pre-F stabilized F protein with HeV G or CedPV G (Figures 6A,B far right panels) may expand the breadth and neutralization potential of the immunogen to multiple virus subtypes and is being tested in ongoing studies. Garg et al., generated two RSV F-PIV3 HN chimeric proteins; F_*R*_HN consisting of RSV F residues 1–529, PIV3 HN residues 87–572) where the RSV F component is in the mostly cleaved, post-F conformation and the other, F_*RipSc*_HN consisting of RSV F in its prefusion conformation (insertion of two mutations N76I and S215P, deletion of the p27 peptide and mutated furin cleavage site) linked with PIV3 HN (104). Cotton rates intramuscularly immunized with F_*R*_HN or F_*RipSc*_HN protein formulated with TriAdj developed high virus neutralizing titers against RSV and PIV3 and prevented RSV and PIV3 replication in the lungs when challenged with RSV or PIV3, respectively (104).

There is global consensus that we are insufficiently prepared for the next pandemic threat by an emerging viral disease. A premise of the prototype pathogen approach (106) is to expand our understanding of the pathogenesis, immunity, and effective vaccine options for representative viruses within each virus family to facilitate the development of interventions for newly emerging viruses. This approach requires identifying generalizable principles that are relevant across viral genera and families. Structure-guided stabilization of surface glycoproteins represents one solution to engineering new or improved candidate immunogens and vaccines that can be broadly applied to paramyxovirus immunogen development. This concept has been applied to the fusion glycoprotein of respiratory syncytial virus (48, 50) and extended to bovine RSV (107) and hMPV (97). It has also been applied successfully for the coronavirus spike glycoprotein (98, 99) and other paramyxoviruses (45).

Here, we have attempted to create a knowledge base of vaccine antigen design concepts for NiV virus as a representative of henipaviruses and informative for other paramyxoviruses. We identified specific mutations that stabilize the NiV F glycoprotein in its pre-F conformation to improve immunogenicity and protein expression levels. We also confirmed that the NiV G glycoprotein is an effective vaccine immunogen and that there are design options to deliver both pre-F and G as a single vaccine construct that confer greater diversity of response to antigenic sites. The information generated from these NiV studies was directly applied to vaccine immunogen designs for two other henipaviruses, Hendra and Cedar viruses. Although directly transferring pre-F stabilizing mutations resulted in relatively well-formed fusion proteins, slightly different combinations worked better for each of the new viruses. Similarly, the chimeric pre-F/G design was applicable to both viruses, but protein expression was lower than for NiV pre-F/G. While there is a crystal structure of the HeV pre-F structure (46), allowing for further direct design to be completed; there is no crystal structure available for Cedar virus pre-F. The structure-based design principles utilized with NiV have been applied to other paramyxoviruses including hPIV3, measles and mumps. While it is possible that G alone is sufficient to protect against all henipaviruses as G is the predominant neutralizing target, the F protein of related paramyxoviruses is often the dominant neutralizing target. Therefore the pre-F/G (HN/H) chimera would theoretically be the lead candidate as a general approach for vaccine development across the paramyxovirus family that could be quickly applied, with limited experimentation, in the event of a pandemic. These studies demonstrate how structure-guided antigen design can be used to rapidly develop vaccine candidates for pandemic threats and supports use of prototype pathogens as an approach to pandemic preparedness and response.

## Data Availability Statement

The datasets generated for this study are available on request to the corresponding author.

## Ethics Statement

All animal experiments were reviewed and approved by the Animal Care and Use Committee of the Vaccine Research Center, NIAID, NIH, and all animals were housed and cared for in accordance with local, state, federal and institute policies in an American Association for Accreditation of Laboratory Animal Care (AAALAC)-accredited facility at the NIH.

## Author Contributions

RL, GS-J, JSM, JRM, and BG designed constructs. RL, GS-J, YT, RC, LK, JRM, and BG designed the research. RL, GS-J, YT, RC, AC, SN, and GH performed the research. RL, GS-J, YT, RC, AC, SN, LK, and BG analyzed and interpreted the data. RL, GS-J, LK, and BG wrote the manuscript. YT, KM, and JSM helped to edit the manuscript.

## Conflict of Interest

YT is employed by Leidos Biomedical Research, Inc., supported in part with funds from the Frederick National Laboratory for Cancer Research, NIH, under contract HHSN261200800001. RL, GS-J, JSM, JRM, and BG are inventors on patent applications involving Nipah virus vaccine designs. The remaining authors declare that the research was conducted in the absence of any commercial or financial relationships that could be construed as a potential conflict of interest.
