# Supplementary material for: Structure-Based Design of Nipah Virus Vaccines: A Generalizable Approach to Paramyxovirus Immunogen Development
Source: Front Immunol. 2020 Jun 11;11:842. doi: 10.3389/fimmu.2020.00842 (PMC7300195; doi:10.3389/fimmu.2020.00842)
Supplement: Supplementary file 1 [file Presentation_1.pdf]

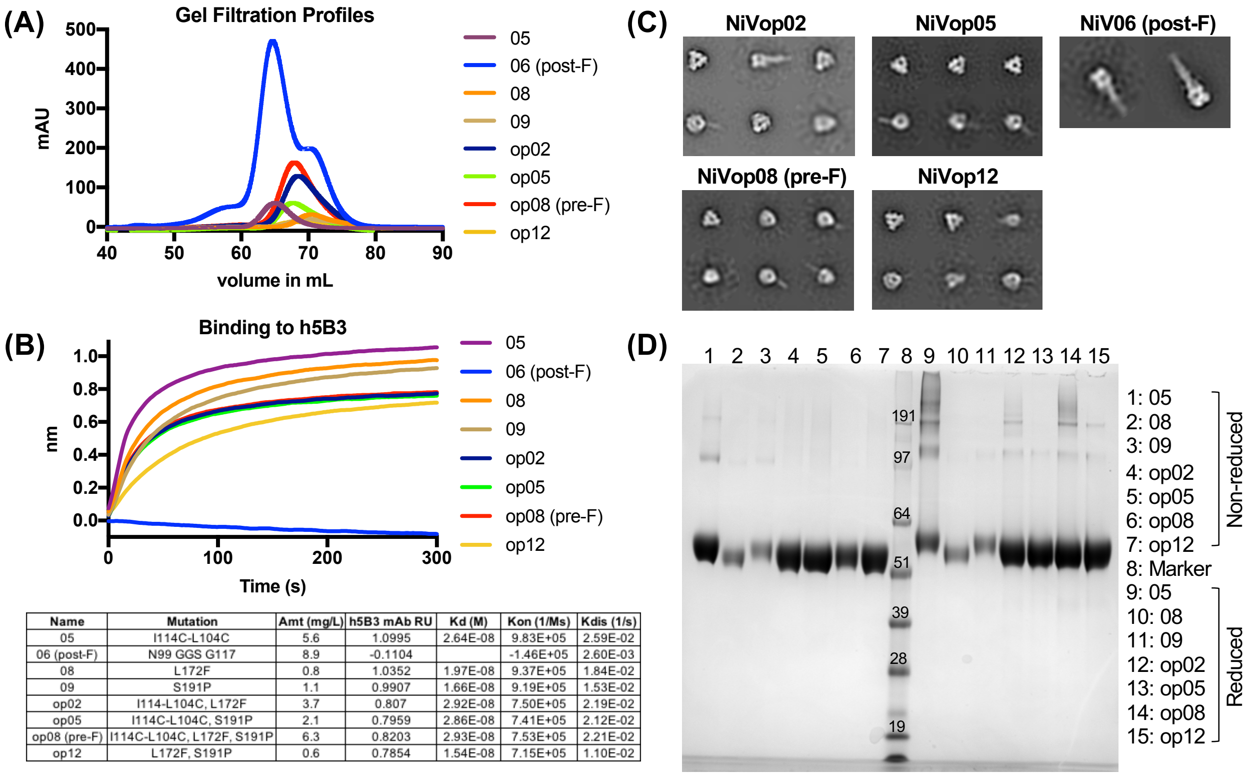

Name	Mutation	Amt (mg/L)	h5B3 mAb RU	Kd (M)	Kon (1/Ms)	Kdis (1/s)
05	I114C-L104C	5.6	1.0995	2.64E-08	9.83E+05	2.59E-02
06 (post-F)	N99 GGS G117	8.9	-0.1104		-1.46E+05	2.60E-03
08	L172F	0.8	1.0352	1.97E-08	9.37E+05	1.84E-02
09	S191P	1.1	0.9907	1.66E-08	9.19E+05	1.53E-02
op02	I114-L104C, L172F	3.7	0.807	2.92E-08	7.50E+05	2.19E-02
op05	I114C-L104C, S191P	2.1	0.7959	2.86E-08	7.41E+05	2.12E-02
op08 (pre-F)	I114C-L104C, L172F, S191P	6.3	0.8203	2.93E-08	7.53E+05	2.21E-02
op12	L172F, S191P	0.6	0.7854	1.54E-08	7.15E+05	1.10E-02

Supplemental Figure 1. Biophysical properties of NiV F variants. (A) SD200 size-exclusion chromatography profiles of 8 NiV F variants, highlighting the monodispersed characteristics of the pre-F (NiVop08, red). **(B)** Binding kinetics of the lead stabilized prefusion conformation F designs to the NiV prefusion-specific antibody, h5B3, as measured by fortéBio Octet Red384 instrument. **(C)** Two-dimensional class averages of stabilized prefusion conformation F designs obtained by negative-stain EM. **(D)** SDS-PAGE, under non-reduced and reduced conditions, showing expression of stabilized prefusion conformation F designs.

Supplemental Figure 2

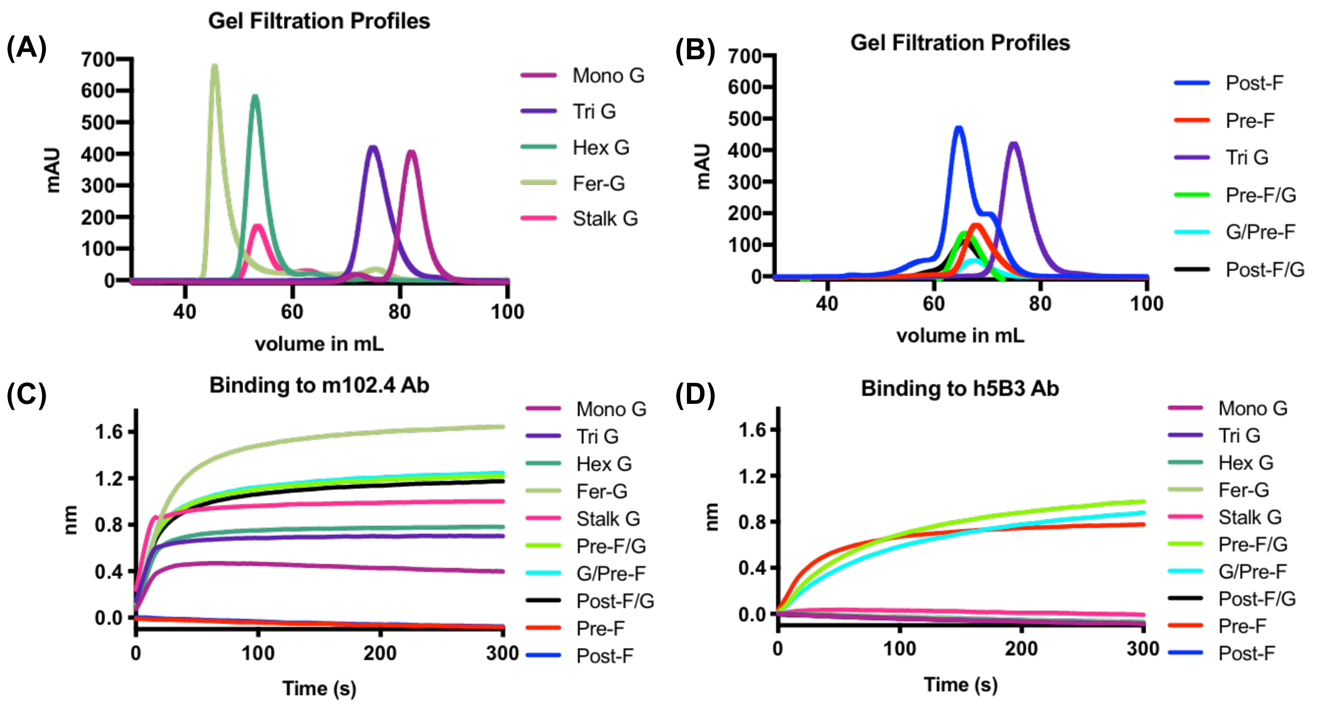

Design	Amt (mg/L)	m102.4 mAb RU	Kd (M)	Kon (1/Ms)	Kdis (1/s)
Mono G	3.8	0.3289	4.02E-07	3.26E+05	1.31E-01
Tri G	1.8	0.7043	1.53E-07	5.59E+05	8.57E-02
Hex G	2.3	0.7873	8.62E-08	6.92E+05	5.96E-02
Fer-G	1.9	1.7017	1.78E-08	1.57E+06	2.79E-02
Stalk G	2.9	1.0189	1.12E-07	7.57E+05	8.49E-02
Pre-F/G	2.0	1.2631	3.10E-08	1.10E+06	3.40E-02
G/Pre-F	0.4	1.3021	2.86E-08	1.12E+06	3.20E-02
Post-F/G	1.4	1.22	3.15E-08	1.07E+06	3.37E-02
Pre-F	6.3	-0.1158		-1.32E+05	2.68E-03
Post-F	8.9	-0.1139		-1.42E+05	2.77E-03

Design	Amt (mg/L)	h5B3 mAb RU	Kd (M)	Kon (1/Ms)	Kdis (1/s)
Mono G	3.8	-0.1286		-1.48E+05	3.04E-03
Tri G	1.8	-0.1217		-1.46E+05	2.95E-03
Hex G	2.3	-0.1136		-1.43E+05	3.01E-03
Fer-G	1.9	-0.1124		-1.54E+05	2.32E-03
Stalk G	2.9	-0.0397		-3.40E+08	1.48E-07
Pre-F/G	2.0	1.1147	9.17E-09	1.05E+06	9.63E-03
G/Pre-F	0.4	1.0204	8.45E-09	9.73E+05	8.22E-03
Post-F/G	1.4	-0.1134		-1.31E+05	2.92E-03
Pre-F	6.3	0.8106	2.88E-08	7.41E+05	2.13E-02
Post-F	8.9	-0.1104		-1.46E+05	2.52E-03

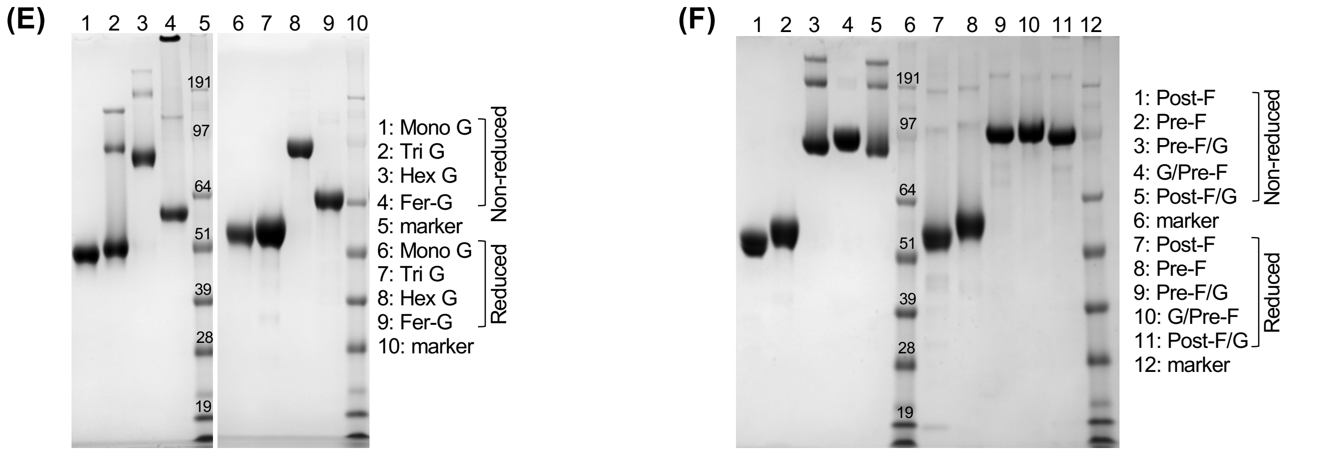

Supplemental Figure 2. Biophysical characterization of NiV G and F/G variants. (A) SD200 size-exclusion chromatography profiles of multimeric forms of G. **(B)** SD200 size-exclusion chromatography profiles of F/G chimeric variants. **(C, D)** Binding kinetics were measured using a fortéBio Octet Red384 instrument and tables summarize binding affinities of multimeric forms of G and F/G chimeric immunogens to the HeV G-specific antibody, m102.4 **(C)** and to the NiV pre-F-specific antibody, h5B3 **(D)**. **(E)** SDS-PAGE, under non-reduced and reduced conditions, showing expression of multimeric forms of G. **(F)** SDS-PAGE, under non-reduced and reduced conditions, showing expression of NiV F/G chimeric designs.

Name	F Amino Acid	Mutation	G Amino Acid	Linker	Trimerization Domain
04	1-488	T101 GSG I114			GCN4
05	1-488	I114C-L104C			GCN4
06 (Post-F)	1-488	N99 GGS G117			GCN4
07	1-488	I114C-I426C			GCN4
08	1-488	L172F			GCN4
09	1-488	S191P			GCN4
op02	1-488	I114C-L104C, L172F			GCN4
op05	1-488	I114C-L104C, S191P			GCN4
op06	1-488	T101 GSG I114, S191P			GCN4
op08 (Pre-F)	1-488	I114C-L104C, S191P, L172F			GCN4
op12	1-488	S191P, L172F			GCN4
op13	1-488	S191P, L172F, Q70G			GCN4
op14	1-488	I114C-L104C, S191P, L172F, Q70G			GCN4
Mono G			172-602		N/A
Tri G			172-602		Fd
Hex G			172-602		Fd
Fer-G			172-602	G_3SG_2	N/A
Stalk G			72-602		N/A
Pre-F/G	1-488	I114C-L104C, S191P, L172F	177-602	GSG_5	GCN4-Fd
G/Pre-F	1-488	I114C-L104C, S191P, L172F	177-602	G_4SG_4	GCN4-Fd

Supplemental Table 1. NiV F, G and Pre-F/G Designs.

Supplemental Figure 3

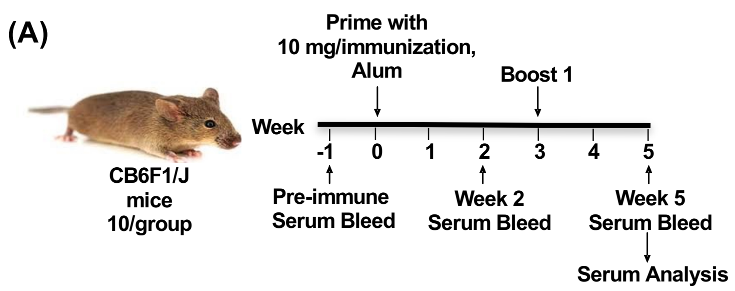

Supplemental Figure 3. Immunogenicity of NiV Pre-F stabilized immunogens, multimeric forms of G and F/G chimeric immunogens. (A) Mouse study immunization scheme. Groups of 10 mice each were immunized intramuscularly with 5 or 10 μg of purified protein, adjuvanted with alum, at week 0 and week 3. Animals were bled at week 2 and week 5. Week 5 sera was analyzed for reactivity and neutralization. **(B,C)** Recognition of pre-F (B) or post-F (C) NiV F proteins by sera from mice immunized twice with NiV F designs or pre-immune sera. **(D, E)** Recognition of pre-F NiV F **(D)** or mono G **(E)** proteins by sera from mice immunized twice with NiV F designs, multimeric forms of G or pre-F/G chimeric immunogens or pre-immune sera. Binding kinetics were measured using a fortéBio Octet Red384 instrument. Line represents mean of all animals in each group +/- standard deviation.

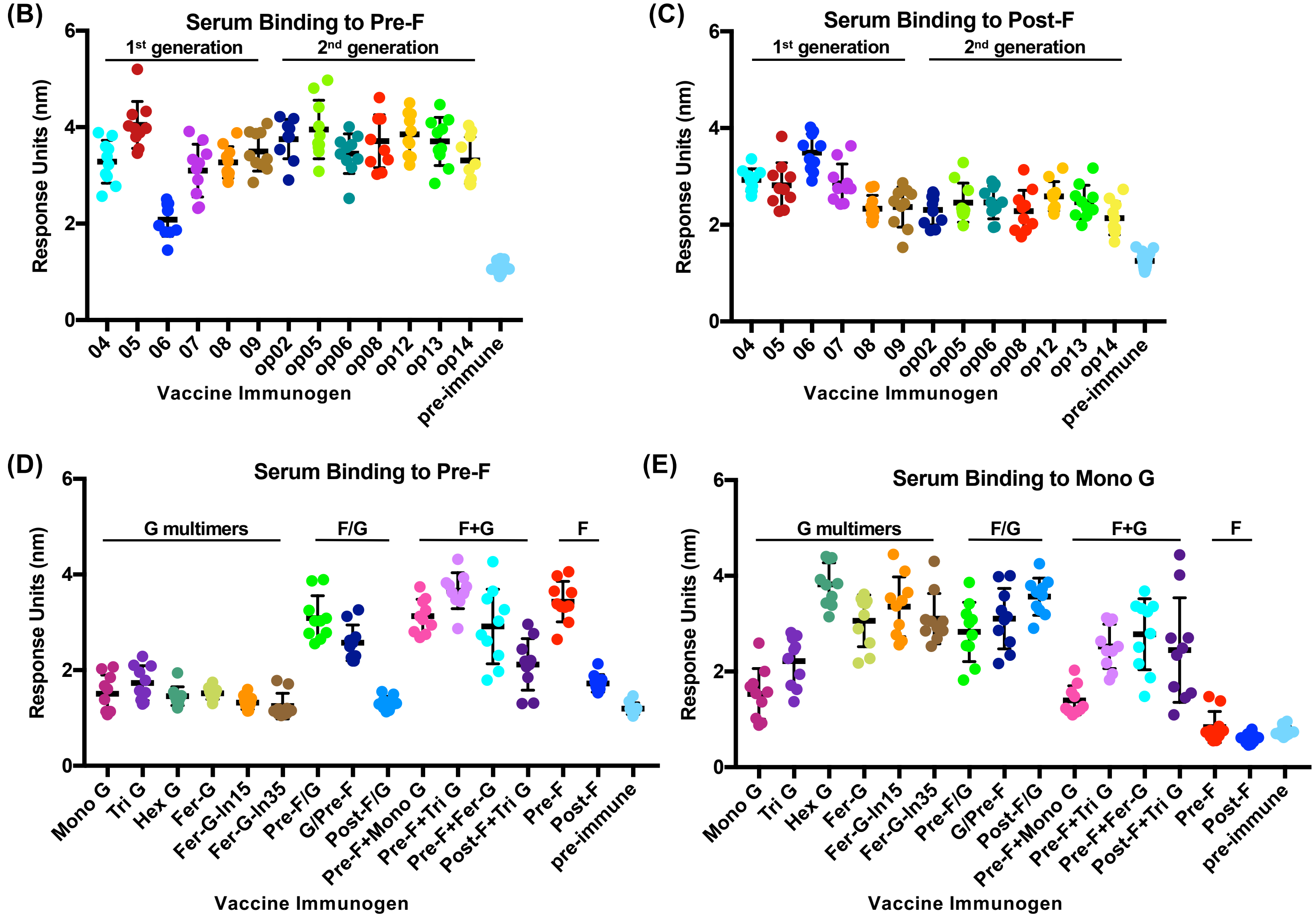

Immunogen	Reciprocal Neutralizing IC80 Titer	
04	639.9	1st generation
05	977.4	
06 (Post-F)	<25	
07	116.8	
08	203.7	
09	1125	
op02	1373	2nd generation
op05	1376	
op06	979	
op08 (Pre-F)	1119	
op12	842	
op13	1534	
op14	290.6	
Mono G	387.5	G multimers
Tri G	628.4	
Hex G	3426	
Fer-G	2103	
Fer-G-In15	3180	
Fer-G-In35	3073	
Pre-F/G	6763	F/G or F+G
G/Pre-F	4535	
Pre-F + Tri G	1406	
Post-F/G	6389	
Post-F + Tri G	1166	

Supplemental Table 2. Neutralization of NiVF/G VSVΔG-luciferase pseudovirus by pooled sera from mice immunized with NiV stabilized pre-F, multimeric G and F/G chimeric immunogens. VSVΔG-luciferase pseudovirus (expresses both NiV F_{WT} and NiV G on surface) neutralization assays were performed on pooled mouse sera collected at week 5. The reciprocal neutralizing IC_{80} titer for each sample was calculated by curve fitting and non-linear regression using GraphPad Prism.
